# Mixed Aqueous-and-Oil Foams via the Spinning Together of Separate Particle-Stabilized Aqueous and Oil Foams

**DOI:** 10.1021/acs.langmuir.1c03348

**Published:** 2022-03-30

**Authors:** Yuchen Si, Tao Li, Paul S. Clegg

**Affiliations:** † School of Physics and Astronomy, University of Edinburgh, Peter Guthrie Tait Road, Edinburgh EH9 3FD, U.K.; ‡ Wenzhou Institute, University of Chinese Academy of Sciences, Wenzhou, Zhejiang 325001, P. R. China

## Abstract

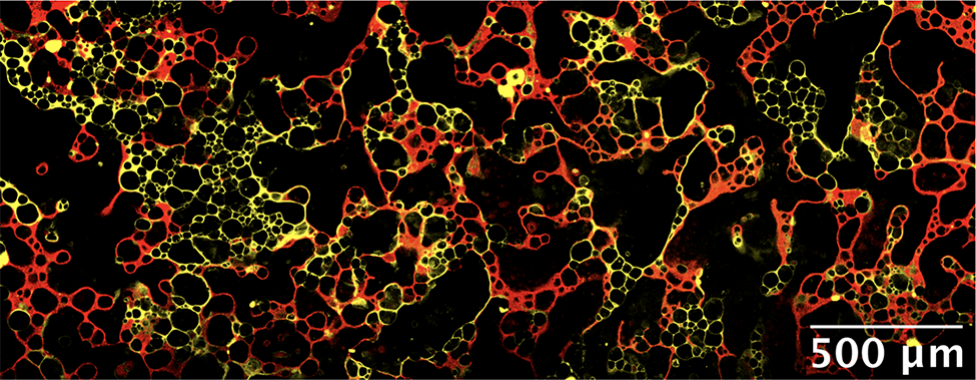

We describe an experimental technique for the production of foams comprised of bubbles in a continuous phase of balanced quantities of aqueous and oil phases. Initially, two highly stable foams are fabricated: one typically made from olive oil with bubbles stabilized using partially fluorinated particles and the other made from a mixture of water and propylene glycol with bubbles stabilized using partially hydrophobic particles. After a rough mixture is prepared, the final mixed foam is fabricated via spinning the components together; the spinning leads to the final foam being well-mixed and dry. Here the final mixed foams are presented in thin-film form. We show the locations and roles of the various components.

## Introduction

Media comprising aqueous and organic components are found throughout foods, personal care products, and home care products.^1,2^ The addition of air provides a route for controlling the density, composition, and flow properties.^3,4^ Furthermore, in material, cleaning, and catalysis applications, combined water–oil–air systems provide properties that cannot be accessed by any other route. To date, such composites have tended to feature one of the two liquids as a minority component^5,6^ or have employed droplets as the dominant component;^4,7,8^ these design strategies can be a distinct disadvantage. Moving beyond these regimes is an important challenge. A foam with balanced quantities of oil and water could be applied to the cleanup of unspecified toxins following a terrorist incident, for example.

Colloidal particles become trapped at liquid–fluid interfaces provided that the particles do not have a substantial affinity for one of the media in preference to the other.^9,10^ Hence, partially hydrophobic particles will become trapped at water–air interfaces, whereas hydrophilic particles would disperse in the water and fully hydrophobic particles would rest on the surface of the water (provided they are not too large and dense).^11^ Once trapped at the interface, partially hydrophobic particles require energies much larger than that of regular Brownian motion to dislodge.^12,13^ With a large population of these particles, bubbles can be stabilized for long periods of time, to give a particle-stabilized foam. The escape of the trapped vapor is suppressed in these foams to a remarkable extent.^14−16^

As described, it is possible to balance the affinity of particles between air and water by carefully adjusting the hydrophobicity of the surfaces. This can be done via, e.g., the addition of methyl groups to the particle surfaces. To stabilize bubbles in an oil, the oleophobicity must be controlled.^17,18^ This can achieved via the use of partially fluorinated particles (or via alternative approaches in edible foams^3,19^). Colloidal particles with varying degrees of fluorination have been used to create long-lived foams from a wide range of oils.^17^ In general, the surface tension of oil–air interfaces is significantly lower than that of water–air interfaces. This means that, while still substantial, the energies required to dislodge particles from oil–air interfaces are comparatively lower.

These differing surface tensions for water–air and oil–air interfaces have been harnessed by Behrens to create novel multicomponent foams.^5,6^ Here small quantities of an oil are added to a particle-stabilized aqueous foam. Due to the surface tension difference, the oil tends to preferentially adsorb to the bubble surfaces without, however, behaving like an antifoam. Furthermore, by carefully tuning the wetting relationship between the oil and the particles, one can form capillary bridges by the oil between the particles leading to the formation of a capillary foam (by analogy to capillary gels^20^).^5^ This study is the first systematic investigation of water–oil particle-stabilized foams of which we are aware.

In this paper, we present a route to creating foams from mixtures of aqueous and oily precursor foams where the two solvents are present in roughly equal quantities. Both of the precursor foams are stabilized by solid particles. We show the optimization of these initial foams and present the final mixed foams in thin-film form.

## Results and Discussion

**Figure 1:**
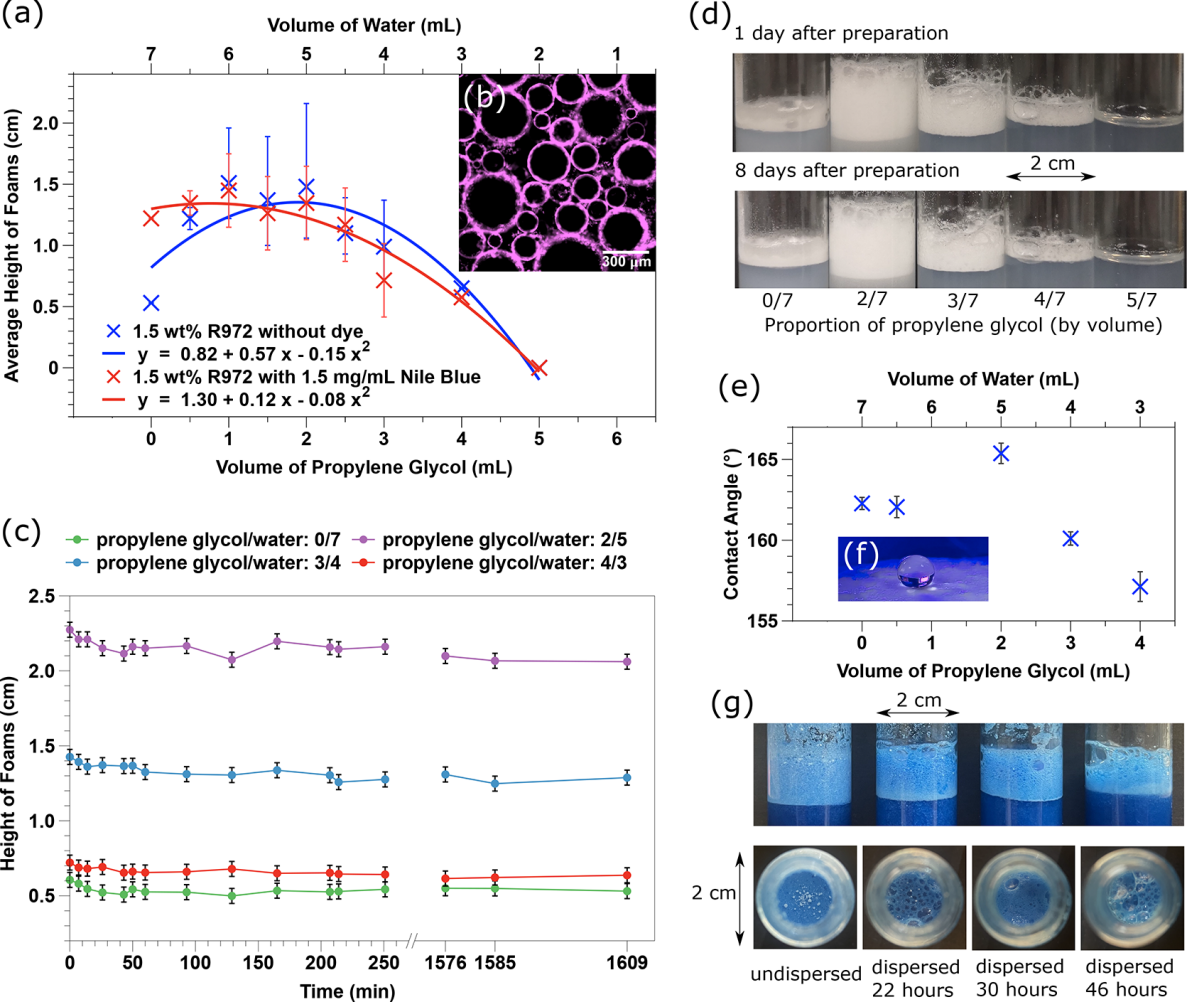
(a) Average heights of aqueous foams (1.5 wt % R972 particles) over 1 day vs the proportion of ultrapure water and propylene glycol. (b) Nile blue-dyed silica particles are attached to the bubble surfaces. (c) Variation of the aqueous foam height for 1.5 wt % particles vs time. (d) Photographs of 1.5 wt % particle-stabilized aqueous foams on days 1 and 8 (volume fraction of propylene glycol listed). (e) Variation of the contact angle of a droplet on a particle bed in air with concentration of propylene glycol (repeated in triplicate). (f) Photograph of the contact angle measurement. (g) Effect of the particle dispersion (1.5 wt % particle-stabilized aqueous foams, 2:5 propylene glycol:water volume ratio). The sample is transformed from undispersed to partly dispersed to well-dispersed to overdispersed.

We begin by optimizing the properties of our aqueous foam. Panels a and d of Figure 1 show that the height of the pure water-based foam increases significantly upon addition of a finite amount of propylene glycol. The addition of propylene glycol makes the aqueous phase less polar. This modifies both the particle–particle interactions in the aqueous phase and the three phase contact angle at the liquid–air interfaces. All of these aqueous foams are stabilized by the Aerosil R972 hydrophobic fumed silica particles (Experimental Section), and the silica particles are located at the bubble interfaces (see Figure 1b). The peak height of an aqueous foam, stabilized by 1.5 wt % silica, is observed when the ratio of propylene glycol and water is around 2:5. As the proportion of propylene glycol increases further, the height starts to decrease. Finally, no foam can be generated when the ratio reaches 5:2. The foam heights decline during the first 30 min after preparation and then remain stable (see Figure 1c). When the concentration of propylene glycol is optimized, the foams persist for >8 months at room temperature. With the same proportion of propylene glycol, the height of the foam increases with silica particle concentration. The variation of the foam height with propylene glycol concentration is echoed by the contact angle of an aqueous droplet on a bed of particles (Figure 1e), although this variation is not that large and also reflects the nature of a fluffy bed of dry particles (Figure 1f). Indeed, the rough surface presented by the undispersed fluffy aggregates is likely to make the contact angle more extreme. Hence, it is expected that the effect of dispersing the fluffy particle aggregates in the solvent changes their wettability markedly. For undispersed and incompletely dispersed samples, a small quantity of bubbles is mingled with particle agglomerations, as one can see in the subphase (see Figure 1g). Large lumps of undispersed particles make the foam less uniform and inhibit our exploration of the relationship between two populations of bubbles. On the microscopic scale, the extent of particle dispersion is a crucial factor during foam preparation. Again, this reflects the relationship between the roughness of a particle cluster (albeit, now on a much smaller scale) and the contact angle. For well-dispersed samples, fine foams are generated. However, if the particles are dispersed for too long, the foam height will decrease and the mean bubble size will increase. Our experience suggests that a 1.5 wt % silica sample needs to be rotated by a rotating lab mixer (speed of 70 rpm) for approximately 30 h for the particles to be ideally dispersed, and each additional 1.0 wt % requires an additional 1 day. This prolonged period required to disperse the particles reflects the challenge of dispersing partially hydrophobic nanoparticles in an aqueous solvent, starting from a dry powder of agglomerates. An alternative method involves using a vortex mixer (speed of 3000 rpm) and an ultrasonic bath alternately for ∼6 h. The particles reach the optimum condition for aqueous foam stabilization due to the combined influence of the propylene glycol and particle dispersion.

**Figure 2:**
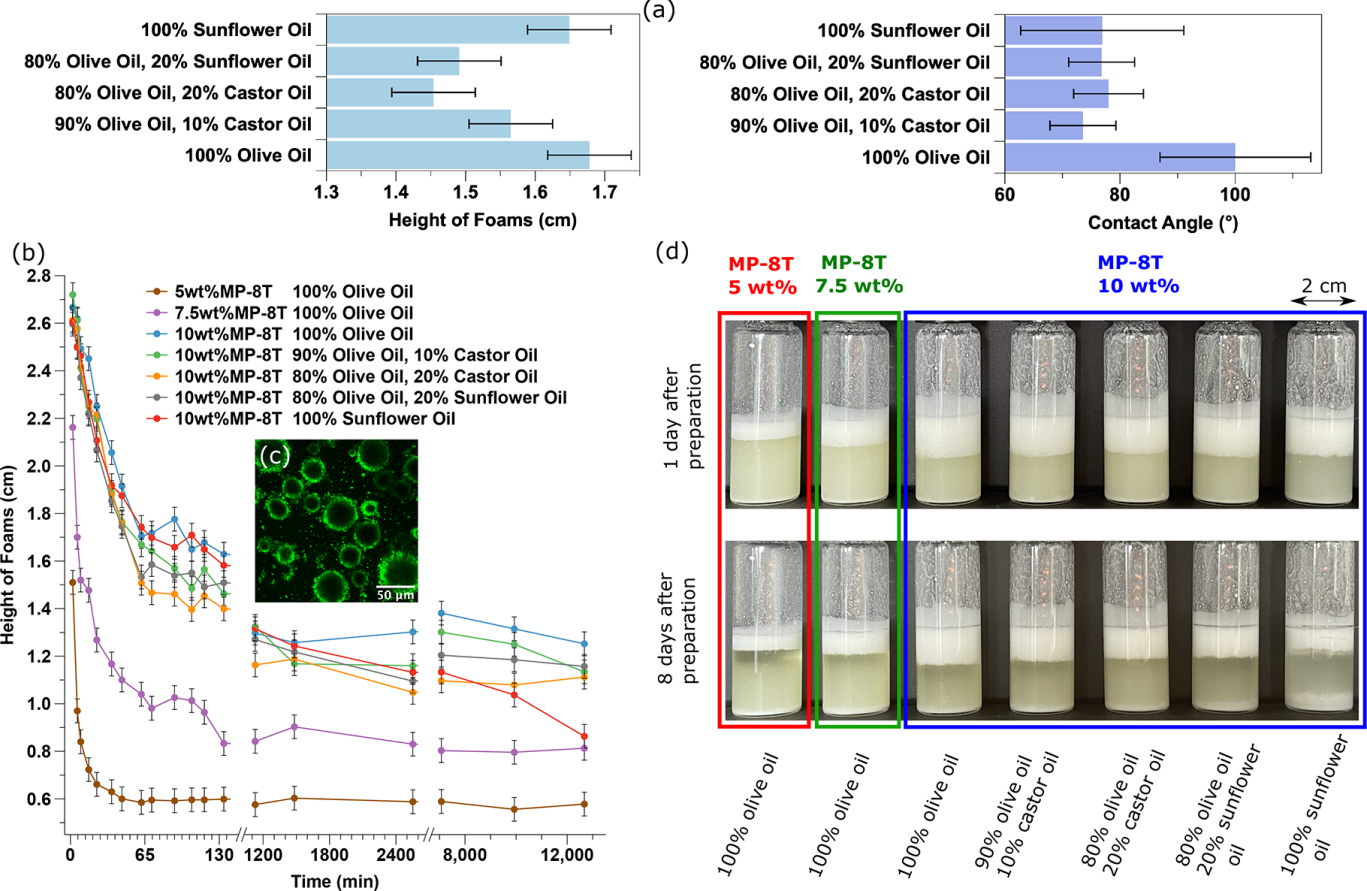
(a) Average heights of oil foam (MP-8T particles) over the first 2 h and the corresponding contact angles for different types of oil. (b) Variation of the height of oil-based foams as a function of time. (c) FITC-dyed MP-8T particles are attached to the bubble surfaces (5 wt % MP-8T/olive oil foam). (d) Photographs for MP-8T-stabilized oil-based foams on days 1 and 8.

Next we turn our attention to the oil foam. The liquid phase of the oil-based foam can be chosen from many kinds of oil. Viscosity is an important parameter for foam formation as a high viscosity (e.g., for castor oil) hinders particle dispersion. However, castor oil can be diluted with other lower-viscosity oils to produce a foam. Additionally, an enhanced viscosity can slow drainage and collisions between bubbles. Figure 2 compares the MP-8T-stabilized olive oil-based, castor oil-based, and sunflower oil-based foams, and the MP-8T particles are located at the bubble interfaces (see Figure 2c). As shown in Figure 2a, the average height of different types of oil-based foam with the same particle concentration shows some variation with oil type over the first 2 h; however, this is scarcely larger than the variation with repeat runs using the same oil. Except for the pure olive oil sample, the particle-oil-air contact angles for all of the other samples are around 75° (see Experimental Section). However, the contact angle for the pure olive oil sample is around 100°. Unlike the aqueous foams, preparing the oil-based foams with MP-8T requires only a few minutes for the oils presented in panels a and b of Figure 2. The particles can readily be dispersed using a vortex mixer leading to stable foams. The lack of stickiness of the particles and the small proportion of residual particles in the continuous phase result in an initial phase of more rapid drainage (Figure 2c), which is not observed for the aqueous foams (Figure 1c). As shown in panels b and d of Figure 2, pure olive oil samples perform best with a slightly larger average foam height. All of the pure olive oil foams with MP-8T concentrations from 5 to 10 wt % are stable for >8 days at room temperature and >8 months when stored in the refrigerator at 4 °C. The oil-based foam with pure sunflower oil is the most unstable, and it starts collapsing from the fifth day after preparation.

**Figure 3:**
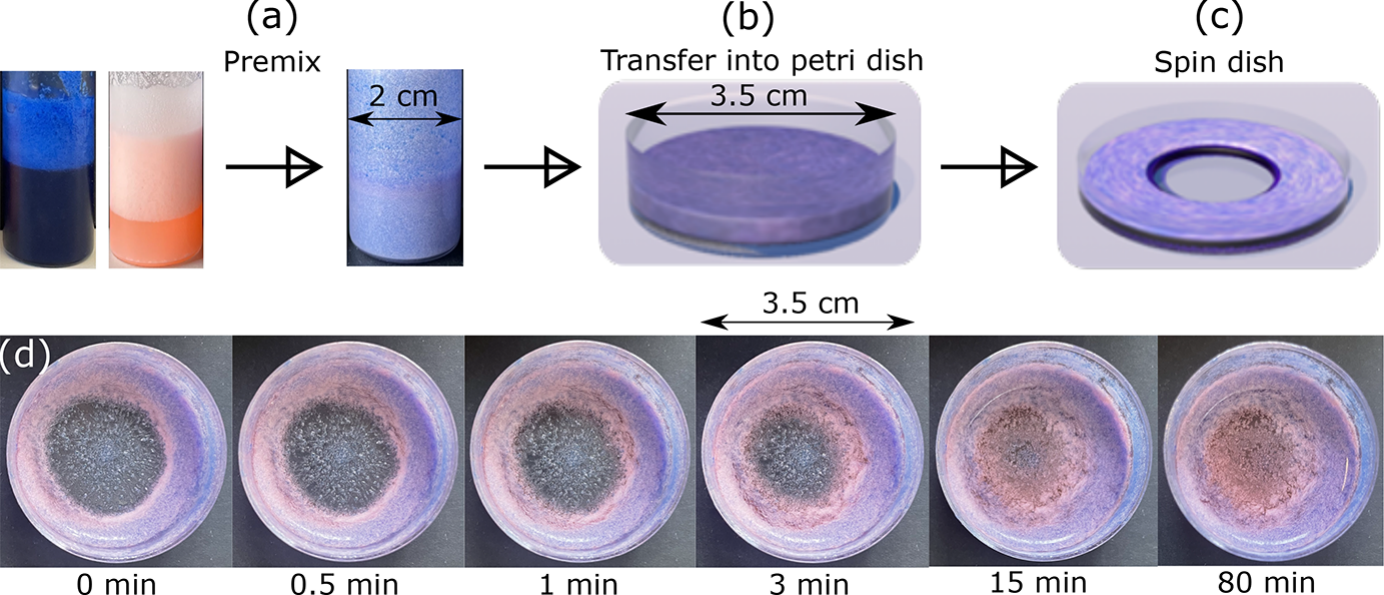
Work flow for composite foam preparation. (a) Premixed oil-based and aqueous foams (photographed here) are transferred into a Petri dish (b) and then spun for 60 s (c) (panels b and c are cartoons). (d) After spinning, a layer of the dryer foam is found floating at the top of an extremely wet foam containing a high proportion of a liquid phase. Photographs show that the dry layer we wish to study will fall back and merge into the bottom wet subphase within a few minutes.

Now we can consider the case of a mixed foam. Panels a–c of Figure 3 are the three steps of mixed foam preparation presented as a flowchart. First, freshly prepared oil-based and aqueous foams are transferred into the same vial for premixing (Figure 3a). As the mass density of the oil-based foams (with a smaller average bubble size and a higher proportion of the continuous phase) is higher than that of the aqueous foams, the aqueous foam should be placed on the top of the oil-based foam to avoid the former being crushed. Premixing the foams by 60 s hand-shaking is an essential step that promotes the mixing of bubbles at the cluster level. Then, the premixed composite foams are transferred into a Petri dish for spinning; the rotation speed, acceleration, and time are set to 2000 rpm, 500 rpm/s, and 60 s, respectively (Figure 3b). These two types of foams do not tend to combine automatically as the oil and water phase repel each other. The turntable of a spin-casting device (see Experimental Section) provides suitable force to promote both liquid drainage and bubble mixing (Figure 3c). Through the spinning process, the foams blend further and simultaneously shed liquid via drainage. After spinning, there is a layer of dryer foam floating at the top of an extremely wet foam that contains a large proportion of the liquid phase (Figure 3c). This dry floating foam layer is the mixed foam that we wish to recover. However, given a few minutes, this floating layer will sink and merge with the lower wet foam. Figure 3d shows photographs of this process during which, we assume that, subphase liquid is drawn back into the spun mixed foam as the bubble shapes begin to relax.

**Figure 4:**
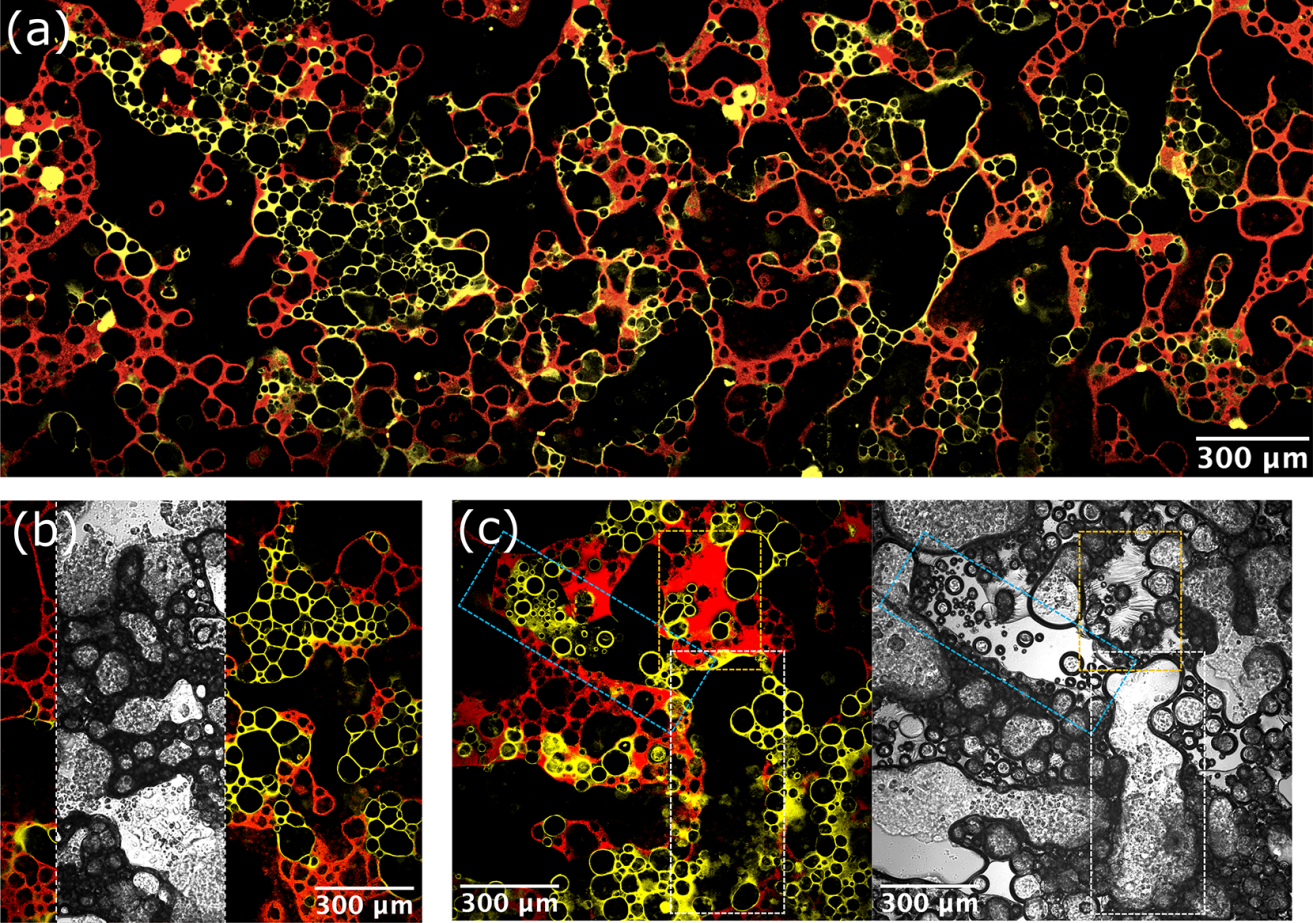
Confocal and bright-field micrographs for composite foams made from yellow aqueous foam (1.5 wt % R972 silica particles, 2:5 propylene glycol:water volume ratio, dyed by Nile blue, false colored for the sake of clarity) and red oil-based foam (10 wt % fluorinated particles MP-8T/olive oil, dyed by Nile red). (a) Thin-film sample fabricated following Figure 3 using at least three parts aqueous foam to one part oil foam. (b) Similar to panel a but with the corresponding bright-field micrograph showing that the black regions are air rather than free solvent. (c) Wetter composite foam in thin-film form. Dashed rectangles: free aqueous liquid region (blue), free oil region (orange), and air region (white).

We visualize these mixed foams in thin-film form. Sandwiched between two coverslips, the mixed foams show different structures due to the relative amounts of oil and aqueous foams and the liquid content (wetness). Space constraints and external forces are provided by locating the sample between the coverslips. This arrangement tends to drive some liquid out of the sample and stabilizes bubbles in a distorted configuration. Figure 4a shows a confocal micrograph for a two-dimensional midwet composite foam. This is the result of combining at least 3 times the volume of aqueous foam (2:7 propylene glycol with 1.5 wt % particles) with oil foam (with 10 wt % particles). The top layer after spinning (in Figure 3c) then has an appropriate liquid fraction. Due to bubble coalescence, the tortuous bubbles act as self-assembled air channels across large parts of the samples. Regions of oil and aqueous phase border some bubbles, and the oil and aqueous phase meet at points along the borders. Figure 4b shows that all of the black regions in this kind of foam at this liquid fraction are gas channels, and a majority of the bubble edges are not circular. It is not always the case that black regions are the gas phase. The confocal and bright-field images for a two-dimensional wet composite foam (Figure 4c) can be contrasted with Figure 4b. A wet sample can be obtained when the top layer from spinning (in Figure 3c) recombines with the bottom wetter part giving a liquid fraction that increases with time. In addition to the gas phase (Figure 4c, in the white dashed rectangle), it contains some free aqueous liquid phase that is undyed (Figure 4c, in the blue dashed rectangle) and some free oil phase (Figure 4c, in the orange dashed rectangle). The clear untextured regions in bright-field images can be recognized as the liquid phases, and the spherical discrete aqueous bubbles are often observed in the undyed free aqueous liquid regions. By contrast, the air channels reveal the drying patterns on the glass surfaces that are visible as a rough texture in the bright-field images.

The mixed foams pictured in bulk in Figure 3a and in thin-film form in Figure 4 are extraordinary. In all previous observations, bubbles within a foam have tended to have been found all within the typical configuration. This could be all bubbles within one phase or all bubbles contacting both phases. Here we observe the case that is clearly well-removed from these two thermodynamically favored cases: the bubbles are simultaneously observed in each phase separately, and some bubbles are observed in contact with both phases. Evidently, the presence of trapped interfacial particles is preventing the composite system from relaxing to one of the favored configurations.

**Figure 5:**
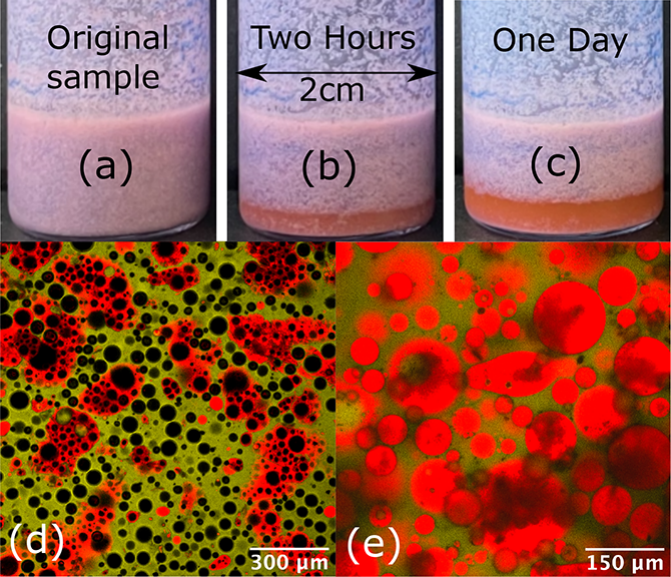
(a–c) Drainage states of the original mixed foam and that after 2 h and 1 day, respectively. (d) Confocal micrograph of a composite foam without a top glass slide with yellow aqueous foam (2.0 wt % R972 silica particles, 2:5 propylene glycol:water volume ratio, dyed by Nile blue, false colored for the sake of clarity) and red oil-based foam (10 wt % MP-8T particles with olive oil, dyed by Nile red). (e) For extremely wet foams, after mixing the foam collapses leading to the formation of an emulsion.

Our mixed foams change due to drainage and can be destroyed during mixing. Panels a–c of Figure 5 show that, while the mixed bulk foam is long-lived, the oil begins draining within hours. Furthermore, while the mixed foam is comprised of both aqueous and oil foams, it is clear from these images that local regions of enhanced pink and blue are present. It seems likely that some local separation is occurring during drainage (Figure 5c). Figure 5d shows a confocal micrograph for a composite foam in bulk (i.e., without a top cover slide). Figure 5e shows a failed foam sample that then forms an emulsion. To avoid the formation of the emulsion associated with the collapse of the foam, it is necessary to avoid transferring a large amount of the liquid subphase into the vial when combining the foams (see Figure 3a).

**Figure 6:**
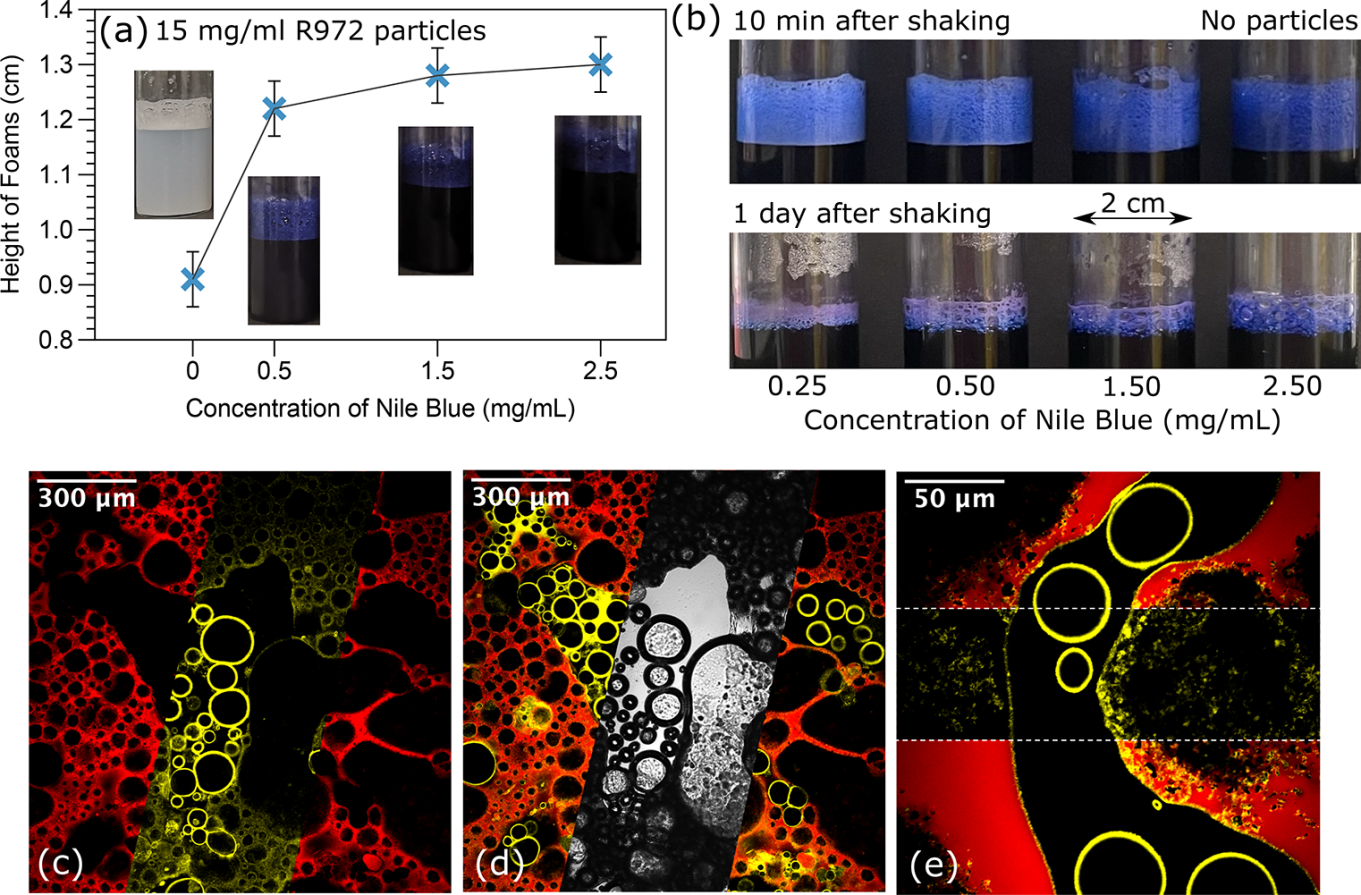
(a) Heights of 1.5 wt % R972-stabilized aqueous foam that increase suddenly with the addition of Nile blue. (b) Aqueous foams stabilized using Nile blue alone after 10 min and 1 day. (c–e) Mosaic images of confocal and bright-field micrographs for composite foams. The yellow aqueous foam (1.5 wt % fumed R972 silica particles in a mixture of propylene glycol to water with a 2:5 volume ratio, dyed with Nile blue, false colored for the sake of clarity) and the red oil-based foam (10 wt % MP-8T particles in olive oil, dyed by Nile red). In panels c and d, the silica particles are trapped at the bubble surfaces. In panel e, the silica particles are attached to the bubble surfaces and the oil–aqueous–liquid interface.

It is an important detail that, in addition to its role as a fluorophore, Nile blue can also be seen to enhance the height of silica-stabilized aqueous foams. Figure 6a shows that the presence of Nile blue significantly increases the foam height; however, there is no significant trend with an increase in dye concentration. The confocal micrographs provide more information about the role of the dye (see Figure 6b). In some foams, typically of midwet composition, the aqueous liquid regions between bubbles are observed to be undyed. Instead, the dye is associated with the silica (R972) particles (see Experimental Section). These particles can be seen at the air–aqueous–liquid and oil–aqueous–liquid interfaces (Figure 6c–e). Evidently, the presence of the dye at the particle surfaces, previously investigated in a similar system,^21^ is improving the stability of the foam as shown in Figure 6a. As a further benefit, the association of the dye with the particles enables us to further study the role of the particles. Upon comparison of panels c and d of Figure 6, a faint yellow curve can be seen at the boundary of an air region and a free aqueous liquid region, which indicates that the silica particles are trapped at the air–aqueous–liquid interface. In Figure 6e, a yellow curve appears at the oil–aqueous–liquid interface. Panels c–e Figure 6 show that the silica particles are trapped at the bubble surfaces. However, in the dryer composite foams without a large amount of free aqueous liquid, the silica particles are primarily observed at the bubble surfaces (data not shown).

## Conclusions

We have presented a route for creating mixed foams from the combination of particle-stabilized oil and water foams. Our approach involves making a premixed foam that is then subjected to spinning. The premix remains a foam for many hours. In thin-film form, the spun foam is found to be an intimate mixture of oil and aqueous foams. Intriguingly, bubbles that have both regions of oil and aqueous phase around their periphery can be found. Our results indicate that the inevitable oil–aqueous interfaces are at least partially covered by surplus silica particles.

## Experimental Section

### Material

Hydrophobic fumed silica particles treated with dimethyldichlorosilane (DDS), Aerosil R972 (specific surface area of 90–130 m^2^ g^–1^, average particle size of 16 nm), for use in stabilizing aqueous foams, were supplied by Evonik Industries. Oleophobic polytetrafluoroethylene (PTFE) particles, Ultraflon MP-8T (specific surface area of 4 m^2^ g^–1^, average particle size of 3 μm), for use in stabilizing oil foams, were supplied by Laurel Products. Propylene glycol (Aladdin) is used to modify the aqueous solvent. Ultrapure water was purified by a Milli-Qintegral 10 reagent water system. The performance of the aqueous solvent, ultrapure water containing various proportions of propylene glycol, was assessed via the foam height and droplet contact angle at an air–particle interface. Olive oil, castor oil, and sunflower oil were supplied by Aladdin and used either separately or as mixtures. Nile blue and Nile red used to dye the aqueous and oil phases, respectively, are produced by Aladdin.

### Foam Preparation and Characterization

To prepare particle-stabilized aqueous foams, R972 particle dispersions were created using a rotating lab mixer with a rotational speed of 70 rpm (MX-RD-Pro, DragonLab). The required rotation time depends on the sample concentration; a 1.5 wt % R972 sample needs approximately 30 h, and each additional 1.0 wt % requires roughly an additional 1 day. For the preparation of particle-stabilized oil-based foams, MP-8T particles were first dispersed into oils by using a vortex mixer operating at ∼2500 rpm (Corning) and an ultrasound bath (Kun Shan Ultrasonic Instruments) alternately for a total of 120 s. Aqueous and oil-based foams were generated by a rapid 60 s hand-shaking of their corresponding particle dispersion.

External forces are required when combining different types of foams that do not automatically mix. Hand-shaking gently (for ∼30 s) provides a suitable force for premixing the composite foams, and spinning can then promote drainage and mixing. The samples are spun using a SYSC-100A Spin Coater (Shanghai SAN-YAN Instrument Co., Ltd.). Photographs of vials and Petri dishes containing samples were taken with either an iPhone 12 (Apple) or a P20 (Huawei). All of the confocal micrographs were taken by the Nikon Eclipse Ti fluorescence microscope. Densities of foams were determined using a graduated cylinder and an analytical balance (Sartorius). The liquid fraction of the foams can be estimated by comparing the density of foams with that of air.

### Contact Angle Measurements

By pressing the particles into the recess on a microscope slide with one circular cavity, one obtains a smooth particle interface. Contact angles of a liquid droplet placed on the particle interface in air were determined using a Theta Flex (Biolin Scientific). The temperature was kept at 20.0 °C. Liquid droplets with varying volumes (2–10 μL) were placed on the air–particle interface. For the hydrophobic R972 interface, droplets are mixtures of ultrapure water and propylene glycol in different proportions. On the oleophobic MP-8T interface, mixed oils (including olive oil, sunflower oil, and castor oil) were investigated. For each sample, the result is the average from four measurements with each measurement being the average of left and right contact angles.
